# *In Vitro* Potency and Preclinical Pharmacokinetic Comparison of All-D-Enantiomeric Peptides Developed for the Treatment of Alzheimer’s Disease

**DOI:** 10.3233/JAD-180165

**Published:** 2018-07-03

**Authors:** Elena Schartmann, Sarah Schemmert, Nicole Niemietz, Dominik Honold, Tamar Ziehm, Markus Tusche, Anne Elfgen, Ian Gering, Oleksandr Brener, Nadim Joni Shah, Karl-Josef Langen, Janine Kutzsche, Dieter Willbold, Antje Willuweit

**Affiliations:** a Institute of Complex Systems, Structural Biochemistry (ICS-6), Forschungszentrum Jülich GmbH, Jülich, Germany; b Institute of Neuroscience and Medicine, Medical Imaging Physics (INM-4), Forschungszentrum Jülich GmbH, Jülich, Germany; c Institut für Physikalische Biologie, Heinrich-Heine-Universität Düsseldorf, Düsseldorf, Germany; d Department of Neurology, Faculty of Medicine, JARA, RWTH Aachen University, Aachen, Germany; e Department of Nuclear Medicine, Universitätsklinikum der RWTH Aachen, Aachen, Germany

**Keywords:** Alzheimer’s disease, amyloid-*β*, D-enantiomeric peptides, pharmacokinetic profile

## Abstract

Diffusible amyloid-*β* (A*β*) oligomers are currently presumed to be the most cytotoxic A*β* assembly and held responsible to trigger the pathogenesis of Alzheimer’s disease (AD). Thus, A*β* oligomers are a prominent target in AD drug development. Previously, we reported on our solely D-enantiomeric peptide D3 and its derivatives as AD drug candidates. Here, we compare one of the most promising D3 derivatives, ANK6, with its tandem version (tANK6), and its head-to-tail cyclized isoform (cANK6r). *In vitro* tests investigating the D-peptides’ potencies to inhibit A*β* aggregation, eliminate A*β* oligomers, and reduce A*β*-induced cytotoxicity revealed that all three D-peptides efficiently target A*β*. Subsequent preclinical pharmacokinetic studies of the three all-D-peptides in wildtype mice showed promising blood-brain barrier permeability with cANK6r yielding the highest levels in brain. The peptides’ potencies to lower A*β* toxicity and their remarkable brain/plasma ratios make them promising AD drug candidates.

## INTRODUCTION

Neurodegenerative diseases caused by the aggregation of misfolded proteins are about to become a threatening risk for our aging society and health care systems. Alzheimer’s disease (AD) is one of the most intensively researched progressive neurodegenerative diseases. More than 20 million people worldwide are currently affected by AD and no curative therapy has been developed in the last 110 years since Alois Alzheimer described the disease. This circumstance manifests the extensive medical need for development of a disease modifying or even a curative treatment of AD.

AD is characterized by three major hallmarks: extracellular deposits or plaques consisting of amyloid-*β* protein (A*β*), intracellular deposits consisting of hyperphosphorylated tau protein, and neurodegeneration. A*β* is natively generated throughout lifetime and able to aggregate, thus forming lower molecular weight soluble A*β* oligomers or insoluble A*β* fibrils that make up the plaques [1]. Since about one decade, researchers entitle soluble oligomers to be the most neurotoxic A*β* species [2]. Both A*β* and tau, with their formation, aggregation, and degradation, are prominent targets in AD drug development [3, 4].

In our laboratory, we identified and developed compounds that specifically and directly eliminate toxic A*β* oligomers. Previously, we described the properties of our lead compound D3 (Table 1). D3 consists of twelve D-enantiomeric amino acid residues and has been identified by mirror image phage display [5–9]. It stabilized A*β* monomers in an aggregation-incompetent conformation thus shifting the equilibrium between A*β* monomers and oligomers away from oligomers toward monomers. D3 eliminated A*β* oligomers *in vitro*, improved cognition and lowered A*β* plaque load in transgenic AD mouse models, and revealed promising *in vivo* properties, e.g., extraordinary high proteolytic stability and beneficial pharmacokinetic characteristics [10–22]. In an optimization approach, D3’s amino acid residue sequence was systematically replaced by natural and unnatural amino acid residues, using peptide microarrays, and screened for their affinity and specificity to monomeric A*β*. The most promising seven peptides (ANK1-ANK7) were selected and further investigated *in vitro* awarding ANK6 the most promising properties [23]. Another optimization strategy to increase the peptides’ efficiency to eliminate toxic A*β* oligomers as well as to increase pharmacokinetic availability was the head-to-tail cyclization of D3 and various derivatives [24, 25]. Additionally, linear tandem 24-mer D3-derivatives in head-to-tail arrangement were designed, pharmacokinetically investigated [26], and successfully tested *in vitro* as well as *in vivo* to reduce symptoms of AD pathology, even more efficiently than the corresponding 12-mer peptides [19, 26, 27].

**Table 1:** Peptides’ sequences and configurations

Peptide	Amino acid residue sequence	Amino acid residue configuration
D3	rprtrlhthrnr-NH_2_	all-D-enantiomeric
ANK6	rkrirlvtkkkr-NH_2_	all-D-enantiomeric
tANK6	rkrirlvtkkkrrkrirlvtkkkr-NH_2_	all-D-enantiomeric
cANK6r	rkrirlvtkkkrr head-to-tail cyclized	all-D-enantiomeric

Here, we further investigated and characterized the most promising microarray-derived derivative ANK6 and compared it to two ANK6-variants, the head-to-tail linear tandem ANK6 (tANK6) and the head-to-tail cyclic ANK6 with an additional arginine (cANK6r) to maintain the total net charge, thus combining different peptide optimization tools. We conducted *in vitro* experiments that exhibit the D-peptides’ impacts on A*β*_1 - 42_ aggregation (A*β* aggregation assay), on A*β* oligomer elimination (QIAD assay), and on A*β*_1 - 42_-induced cytotoxicity (cell viability assay). In another pre-*in vivo* test, the D-peptides’ plasma protein binding affinities were determined. Thereby, we explored whether ANK6 and its two variants are efficient and non-toxic A*β*-targeting AD drug candidates. Afterwards, pharmacokinetic studies with intravenous (i.v.) and oral (p.o.) D-peptide administration to C57BL/6N mice were carried out to evaluate the D-peptides’ eligibility for implementation in further treatment studies. Within the pharmacokinetic studies, special attention was paid to the D-peptides’ uptake into the brain and their blood-brain barrier (BBB) permeability.

## MATERIALS AND METHODS

### Peptides

Non-labelled peptides ANK6, tANK6r, and cANK6r were synthesized by peptides & elephants GmbH (Germany). Radio-labelled ^3^H-ANK6, ^3^H-tANK6, and ^3^H-cANK6r were produced by Cambridge Research Biochemicals (United Kingdom) with 1 mCi/mL. The peptides’ sequences are shown in Table 1. Recombinant A*β*_1 - 42_ was obtained from Isoloid GmbH (Germany). Synthetic A*β*_1 - 42_ was obtained from Bachem AG (Switzerland).

### D-peptides’ in vitro potencies

#### A*β* aggregation assay

The potencies of ANK6, tANK6, cANK6r, and D3 to inhibit A*β*_1 - 42_ aggregation was examined using Thioflavin T (ThT). For this purpose, a buffer solution composed of 20 mM sodium phosphate buffer (pH 7.4) including 50 mM sodium chloride, and 5*μ*M ThT was prepared. Afterwards, ANK6, tANK6, cANK6r, or D3 were diluted in this solution, respectively, to final peptide concentrations between 0.3125 and 80*μ*M (ANK6 and tANK6:0.3125, 0.625, 1.25, 2.5, 5, 10, 20*μ*M; cANK6r: 1.25, 2.5, 5, 10, 20, 40, 80*μ*M; D3:1.17, 2.34, 4.7, 9.4, 18.8, 37.5, 75*μ*M).

The buffer solution only served as negative control (blank). Lyophilized A*β*_1 - 42_ was dissolved in the respective peptide solution to a final concentration of 10*μ*M. D-peptide solutions with the highest concentration of the respective D-peptide containing all components, except of A*β*_1 - 42_, served as negative controls. A*β*_1 - 42_ solved in buffer solution without D-peptide served as positive control. 100*μ*L of each solution were transferred into the wells (triplicates) of 96 well microplates (PS, black, non-binding from Greiner Bio-One, Germany) and fluorescence signals (excitation*λ* 440 nm, emission*λ* 490 nm) were measured in a Polarstar Optima plate reader (BMG, Germany) at 37°C every 20 min for 70 h. Afterwards, the respective blank curves were subtracted from each aggregation curve to exclude background fluorescence. The inhibition potency of each D-peptide’s concentration was calculated by normalizing the final fibril mass to the positive control, which shows maximal A*β* aggregation and therefore maximal fluorescence signals (0% A*β* aggregation inhibition). The half maximal inhibitory concentration (IC_50_ value) was determined by plotting the percent inhibition against the respective D-peptides’ concentrations. Datasets were fitted by nonlinear regression (one site – specific binding with Hill slope, GraphPad Prism 5).

#### Cell viability assay

The ability of ANK6, tANK6, and cANK6r to neutralize the toxicity of oligomeric A*β*_1 - 42_ was investigated with 3-(4,5-Dimethylthiazol-2-yl)-2,5-diphenyltetrazoliumbromid (MTT). In this experiment, we used rat phaeochromocytoma cells (PC12 cells, Leibniz Institute DSMZ, Germany) cultivated in DMEM supplemented with 10% fetal calf serum, 5% horse serum, and 1% penicillin-streptomycin at 37°C, 5% CO_2_, and 95% humidity. The cells (10,000 cells/well) were incubated on collagen coated 96 well plates (Gibco, Life Technologies, # A11428-03) for growth in adherent cell culture (24 h, 37°C). Oligomeric A*β* was generated by incubating monomeric A*β*_1 - 42_ in sodium phosphate buffer (10 mM Na_2_HPO_4_/NaH_2_PO_4_, pH 7.4) at 21°C and 600 rpm agitation for 4.5 h. The cell viability was investigated after the incubation with buffer only (positive control, set to 100 % cell viability), Triton X-100 (0.125 %, cytotoxic agent, negative control), A*β*_1 - 42_ alone (1*μ*M), ANK6, tANK6, or cANK6r alone (15*μ*M each), as well as A*β*_1 - 42_ (1*μ*M) in the presence of ANK6, tANK6, or cANK6r (each D-peptide in 7 different concentrations varying between 0.008 and 15*μ*M, in quintuplicates) (overnight, 37°C) using the Cell Proliferation Kit I (MTT) according to the manufacturer’s protocol (Roche, Switzerland). Absorbance readout was detected with a Polarstar Optima plate reader (BMG, Germany) at 570 and 660 nm. The relative cell viability [%] was calculated by normalizing the absorbance readout to the positive control (PC12 cells incubated with buffer). The IC_50_ value was determined by plotting the relative cell viability [%] against the respective D-peptides’ concentrations. Datasets were fitted by nonlinear regression (logistic fit; OriginPro 9.0G).

#### Quantitative determination of interference with A*β* aggregate size distribution

As soluble A*β* oligomers are currently expected to be the most neurotoxic A*β* species causing AD, the A*β*_1 - 42_ oligomer elimination potency of ANK6, tANK6, and cANK6r was investigated by the quantitative determination of interference with A*β* aggregate size distribution (QIAD) similar as described before [19]. In short, lyophilized A*β*_1 - 42_ was dissolved in sodium phosphate buffer to a final concentration of 80*μ*M and incubated for 2.5 h (21°C, 600 rpm) to achieve an A*β* aggregate distribution including monomers, oligomers, and higher molecular aggregates. Then, sodium phosphate buffer (control), 20*μ*M ANK6, 20*μ*M tANK6, or 20*μ*M cANK6r were added and incubated for further 40 min (21°C, 600 rpm). Afterwards, the samples were loaded on top of a density gradient (5 to 50% (w/v) iodixanol, OptiPrep, Axis-Shield, Norway) and ultra-centrifuged for 3 h (4°C, 259.000× *g*, Optima TL-100, Beckman Coulter, USA). In the following, 14 fractions (140*μ*L each) were taken from top to bottom, whereby the top fractions (1-2) contained A*β* monomers, the middle fractions (4–6) contained the A*β* oligomers of special interest, and the bottom fractions (11–14) contained high molecular weight co-precipitates. The left-over was diluted in 60*μ*L 6 M guanidine hydrochloride (fraction 15). Finally, the A*β*_1 - 42_ concentrations in all fractions were determined via analytical RP-HPLC (reversed phase-high performance liquid chromatography) and UV absorbance detection at 214 nm.

#### Statistical calculation

Statistical analyses were performed using GraphPad Prism 5 (GraphPad Software, Inc., USA) and SigmaPlot Version 11 (Systat Software, Germany). Gaussian distribution was analyzed by use of a normal probability plot (SigmaPlot or InVivoStat by Simon Bate and Robin Clark, United Kingdom) [28]. Data is represented as mean±SEM, *p* > 0.05 was considered to be not significant. Data was analyzed by one-way ANOVA with Bonferroni *post hoc* analysis.

### Preclinical pharmacokinetic characterization

#### Plasma protein binding

Plasma protein binding (PPB) to human serum albumin (HSA) and *α*1-acid glycoprotein (AGP) of ANK6, tANK6, and cANK6r was analyzed according to the manufacturer’s protocol of TRANSIL^XL^ HSA and AGP binding kits (Sovicell GmbH, Germany). To cover a larger range of HSA and AGP concentrations, the bead concentrations in the kit were modified. For detection, a mixture of ^3^H-labelled and non-labelled ANK6, tANK6, and cANK6r (final concentrations of 5*μ*M) was added to different concentrations of HSA (7.4*μ*M to 420*μ*M, 10–12 different concentrations) or AGP beads (0.04*μ*M to 3*μ*M, 9–12 different concentrations). The amount of unbound ANK6, tANK6, and cANK6r (in %) to HSA or AGP, respectively, was determined by liquid scintillation counter (LSC) measurements. The dissociation constants (K_D_) as well as the free drug fractions (f_u_) in human plasma were calculated as described before [29]. Calculations were based on peptide concentrations detected in the blood 4 h after single oral administration of 10 mg/kg: 0.01*μ*M ANK6, 0.01*μ*M tANK6, and 0.01*μ*M cANK6r.

#### Animals

The pharmacokinetic profiles were investigated in 180 male C57Bl/6N mice aged 13-14 weeks, weighing about 27.4 g in average. Mice were ordered at Charles River (Germany) and housed at least one week under standard housing conditions (12/12 h light-dark cycle, approximately 22°C room temperature and 54% humidity; water and food available *ad libitum*) in the animal facility of the Forschungszentrum Jülich GmbH before the experiments were carried out. All animal experiments were approved by the Animal Protection Committee of the local government according to the German Protection of Animals Act (LANUV, North-Rhine-Westphalia, Germany, Az.84-02.04.2017.A029).

#### Pharmacokinetic concentration-time profiles

To determine the concentration-time profiles of ANK6, tANK6, and cANK6r in murine brain, plasma, liver, kidney, and cerebrospinal fluid (CSF) after i.v. and p.o. administration, mixed solutions of non-labelled and ^3^H-labelled peptides were prepared. The administered solutions contained 1 mg/mL (i.v.) or 3 mg/mL (p.o.) of the respective D-peptide including small amounts of ^3^H-labelled peptides (0.54*μ*g/mL ^3^H-ANK6, 1.79*μ*g/mL ^3^H-tANK6, or 0.98*μ*g/mL ^3^H-cANK6r). Doses were administered by body weight: i.v. 3.3 mg/kg, ANK6 & tANK6 p.o. 10 mg/kg, cANK6r p.o. 15 mg/kg. Three male C57Bl/6N mice aged 13-14 weeks were investigated per time point whereby individual outliers were excluded from further evaluation. Organs were harvested as follows: i.v. 5, 10, 30, 60, 120, 240, 360, 1080, 1440 min; p.o.: 10, 20, 30, 60, 120, 240, 360, 1080, 1440 min.

Animals whose CSF was not extracted were anesthetized with isoflurane (cp-pharma, Germany) inhalation approximately 2 min before each organ harvesting time point. Afterwards, blood was taken by cardiac puncture and the heparinized blood was centrifuged (3000 g, 5 min, 4°C) to get plasma. The plasma in the supernatant was separated and 1:1 diluted with PBS. The right brain hemisphere, 200 mg of the big liver lobe, and the right kidney were removed, weighed, and homogenized in 500*μ*L PBS (Precellys Ceramic Kit 1.4 mm, Precellys 24, Bertin technologies SAS, France). 100*μ*L of the diluted plasma, the homogenized brain, liver, or kidney (in triplicates), or 1–5*μ*L (exactly determined) of the extracted CSF (extraction procedure see below, single determination) were mixed with 10 mL scintillation cocktail (Ultima Gold XR, PerkinElmer, USA). The mixture was incubated overnight (100 rpm, room temperature).

Animals whose CSF was extracted (organ harvesting time points: 60 min, 240 min, 1440 min) were i.p. anesthetized with ketamine/medetomidine approximately 20 min before each sampling time point. When the mouse was in deep narcosis, the *cisterna magna* was laid free and punctuated with a small capillary to extract about 5*μ*L of CSF as described before [30]. Afterwards, cardiac puncture, organ extractions, and sample preparations were performed as described above.

Quantification of the amount of ^3^H-labelled D-peptides was performed with a LSC and the results (dpm/sample) were converted into mg/mL or % injected dose (% ID)/mL for plasma and for CSF, or in mg/g or % ID/g for brain, liver, and kidney as described before [29]. Total peptide concentrations in the samples were back-calculated from the measured ^3^H-labelled peptides’ radioactivity as described before [20, 25, 26, 29].

The determined peptide concentrations were plotted over time to allow for comparison of all peptides’ uptake into brain, plasma, liver, kidney, and CSF. Concentrations at 0 min were set to 0 % ID/mL or 0 % ID/g except for plasma concentrations after i.v. administration. There, concentrations were linearly back-extrapolated based on the first two measured time points (5 min, 10 min).

#### Pharmacokinetic parameters

To calculate the pharmacokinetic parameters of ANK6, tANK6, and cANK6r for plasma and brain, concentration-time profiles were analyzed. Non-compartmental data analysis was performed with Phoenix WinNonlin (Pharsight, a Certara Company; USA) to calculate the area under the curve from the first to the last measured data pair (AUC_last_), the mean residence time (MRT), and the terminal elimination rate constant (*λ*_z_, nonlinear regression of the last four to five measured concentrations). Further pharmacokinetic parameters were calculated with the help of the formulas listed in Table 2.

**Table 2:** Formulas for calculation of pharmacokinetic parameters and blood-brain barrier values

pharmacokinetic parameter		Unit	Formula
t_1/2_	terminal half-life	h	t_1/2_ = ln(2)/*λ*_z_
D	dose	mg/kg
F_AUC%ID_	bioavailability	%	$F_{{\mathrm{AUC}}_{\%\mathrm{ID}}}\frac{{\mathrm{AUC}}_{{\%\mathrm{ID}}_{e.v.}}}{{\mathrm{AUC}}_{{\%\mathrm{ID}}_{\mathrm{tv}.}}}$
CL	terminal plasma clearance	mL/(min*kg)	CL =*λ*_z_*V_last_

blood-brain barrier value		Unit	formula
logBB	blood-brain equilibrium distribution	–	logBB = log(AUC_last, br_/AUC_last, pl_)
K_in_	unidirectional influx rate constant	mL/(g*min)	$\frac{C_{b}(t)}{C_{p}(t)}=K_{\mathrm{in}}*\frac{{\mathrm{AUC}}_{p}(t)}{C_{p}(t)}+V_{i}$
V_i_	initial distribution volume	mL/g	see formula for K_in_
PS	permeability surface-area product	mL/(g*min)	PS = (-CBF)*ln(1-K_in_/CBF)
CBF	(murine) cerebral blood flow	mL/(g*min)	1.07 [32]

To allow for direct comparison with other peptides, four universally applied BBB parameters were determined [31]: the blood-brain equilibrium distribution (logBB), the universal influx rate constant (K_in_), the initial distribution volume in brain (V_i_), and the permeability surface-area product (PS). Based on data of the concentration-time profiles and pharmacokinetic parameters after i.v. administration, the BBB parameters were calculated with the help of the formulas listed in Table 2. Graphical determination of K_in_ and V_i_ was conducted by plotting the brain concentration to plasma concentration ratio at certain time points (C_b_ (t)/C_p_ (t) [mL/g]) on the y-axis against the exposure time (AUC_p_ (t)/C_p_ (t) [min]) on the x-axis. The linear range for K_in_ and V_i_ determination was between 0 and 240 min for ANK6 and cANK6r (R^2^: 0.9970, 0.9996) and between 0 and 1440 min for tANK6 (R^2^: 0.9758). PS was calculated on the basis of a murine cerebral blood flow (CBF) of 1.07 mL/(g*min) [32].

## RESULTS

### D-peptides’ in vitro potencies

**Fig. 1.**
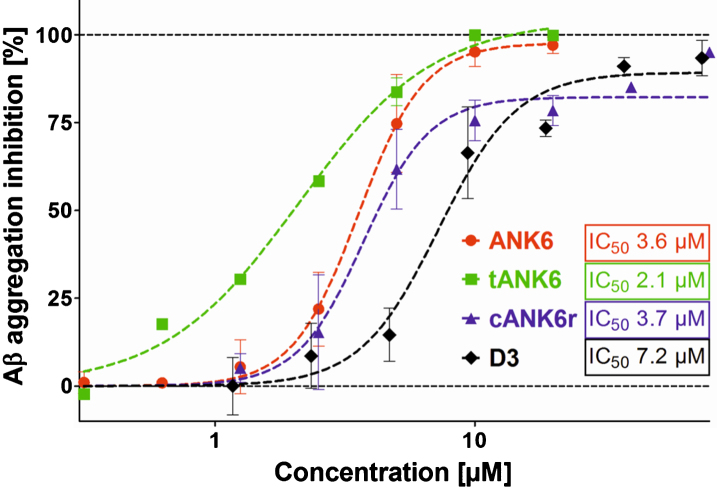
A*β* aggregation assay. To investigate ANK6’s (red circles), tANK6’s (green squares), cANK6r’s (blue triangles), and D3’s (black diamonds) potencies to inhibit A*β*_1 - 42_ monomer aggregation into ThT-positive fibrils, different concentrations of the respective peptides (0.3125-80*μ*M) were incubated with 10*μ*M A*β*_1 - 42_ each. The A*β* aggregation inhibition [%], relative to the fluorescence signal of ThT-positive A*β*_1 - 42_ fibrils which were formed without any peptide added, was plotted against the respective D-peptides’ concentrations. The IC_50_ values were determined by nonlinear regression.

The A*β* aggregation assay was performed to compare the potencies of ANK6, tANK6, and cANK6r, with D3’s potency to inhibit the formation of ThT-positive A*β*_1 - 42_ fibrils. In Fig. 1, the A*β* aggregation inhibition [%], relative to A*β*_1 - 42_ aggregation without peptide, was plotted against different ANK6, tANK6, cANK6, or D3 concentrations (0.3125-80*μ*M). The data fits resulted in the following IC_50_ values: 3.6*μ*M ANK6, 2.1*μ*M tANK6, 3.7*μ*M cANK6r, 7.2*μ*M D3. Equimolar concentrations of ANK6 relative to A*β*_1 - 42_ as well as of tANK6 relative to A*β*_1 - 42_ reduced the aggregation amplitude by more than 97%, while cANK6r and D3 needed about 8-fold molar excess relative to A*β*_1 - 42_ to reduce the aggregation amplitude by more than 95%.

**Fig. 2.**
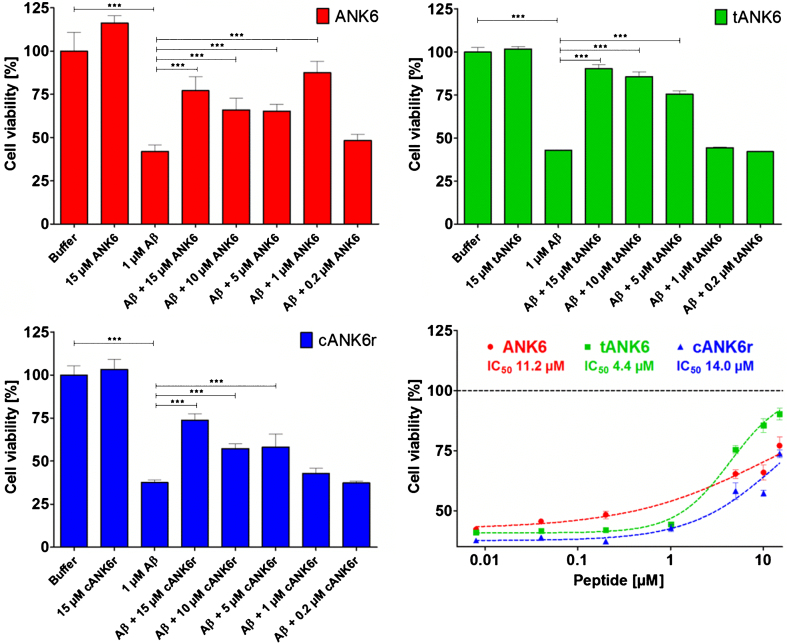
Cell viability assay. To investigate ANK6’s (red circles), tANK6’s (green squares), and cANK6r’s (blue triangles) potencies to reduce the toxicity of A*β*_1 - 42_, a cell viability assay was performed. After pre-incubation of A*β*_1 - 42_ monomers to ensure A*β* oligomerization, solutions containing either A*β*_1 - 42_ alone (1*μ*M final concentration), or A*β*_1 - 42_ with different amounts of ANK6, tANK6, and cANK6r (final concentrations between 0.008 and 15*μ*M) were incubated on PC12 cells overnight. Cell viabilities [%], relative to buffer-treated cells, were plotted against the respective D-peptides’ concentrations. Datasets were fitted by nonlinear regression to determine the IC_50_ values. Data is represented as mean±SEM; one-way ANOVA, ^***^p≤0.001.

To find out whether ANK6, tANK6, and cANK6r lowered the cytotoxic effect of A*β*_1 - 42_ on the cell viability of PC12 cells, MTT assays were performed with various D-peptide concentrations (Fig. 2). In a 1:5 ratio of A*β*:D-peptide, ANK6, tANK6, or cANK6r could hold up the cell viability to 65.3%, 75.5%, or 58% as compared to the cell viability in buffer. Previously published data revealed that D3 could hold up cell viability to about 60% in the same A*β*:D-peptide ratio [23]. For each evaluated D-peptide, we observed a significant reduction of the A*β*-induced cytotoxicity from 15 down to 5 respectively 1*μ*M D-peptide (ANK6: one-way ANOVA F_ (7,39) _ = 77.088 Bonferroni *post hoc* analysis 1:15, 1:10, 1:5, 1:1 (A*β*:ANK6) all *p*≤0.001; tANK6: one-way ANOVA F_ (7,39) _ = 203.781, Bonferroni *post hoc* analysis 1:15, 1:10, 1:5 (A*β*:tANK6) all *p*≤0.001; cANK6r: one-way ANOVA F_ (7,39) _ = 179.813, Bonferroni *post hoc* analysis 1:15, 1:10, 1:5 (A*β*:cANK6r) all *p*≤0.001).

The D-peptides alone did not show negative influence on the cell viability. By plotting the cell viability [%] against the different ANK6, tANK6, or cANK6 concentrations, the following IC_50_ values were determined: 11*μ*M ANK6, 4.4*μ*M tANK6, 14*μ*M cANK6r (Fig. 2).

**Fig. 3.**
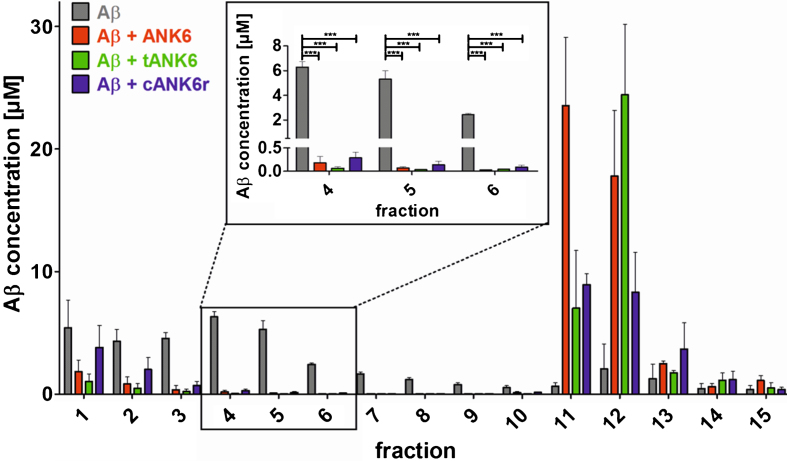
Quantitative determination of interference with A*β* aggregate size distribution (QIAD) assay. To investigate the D-peptides’ impact on A*β* oligomer elimination, a QIAD assay was performed. A*β*_1 - 42_ was pre-incubated to form the natural A*β* aggregate size distribution. Afterwards, sodium phosphate buffer (control, grey bars), ANK6 (red bars), tANK6 (green bars), or cANK6r (blue bars) was added. The samples were separated via density gradient centrifugation into 15 different fractions containing different A*β* particle sizes (fractions 1-2: monomers, 4–6: oligomers, 11–14: high molecular weight co-precipitates). Results revealed that ANK6, tANK6, and cANK6r significantly lowered the A*β* oligomer concentrations in fractions 4 to 6 as compared to the control (A*β* alone). Data is represented as mean±SEM; one-way ANOVA, ^***^p≤0.001.

To investigate the D-peptides’ impact on A*β* oligomer elimination, a quantitative determination of interference with A*β* aggregate size distribution (QIAD) assay was performed as described in the methods section. The results showed that ANK6, tANK6, and cANK6r significantly lowered the A*β* oligomer concentrations in fractions 4 to 6 which contain oligomeric A*β* [19] (fraction 4 one-way ANOVA F_ (3,11) _ = 176.336, A*β*_1 - 42_ versus ANK6, tANK6, or cANK6r *p*≤0.001, fraction 5 one-way ANOVA F_ (3,11) _ = 54.555, A*β*_1 - 42_ versus ANK6, tANK6, or cANK6r *p*≤0.001, fraction 6 one-way ANOVA F_ (3,11) _ = 356.147, A*β*_1 - 42_ versus ANK6, tANK6, or cANK6r *p*≤0.001) (Fig. 3). While cANK6r only slightly lowered A*β* concentrations in fractions 1 and 2 that contain A*β* monomers, linear ANK6 and tANK6 reduced the A*β* monomer concentrations much stronger. As a consequence, fractions 11 and 12, which contain amorphous high molecular weight A*β* co-precipitates with the respective compound, showed higher A*β* content after incubation with ANK6 and tANK6 as compared to cANK6r.

### Preclinical pharmacokinetic characterization

**Fig. 4.**
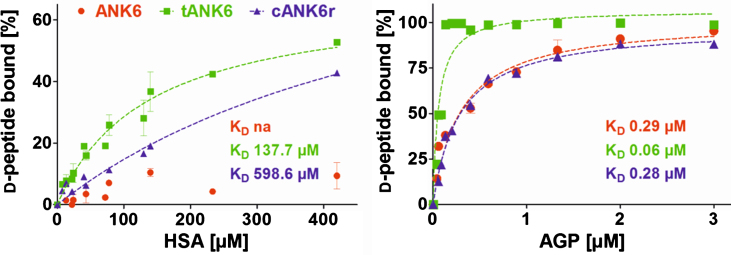
Binding to the plasma proteins HSA and AGP. ANK6’s (red circles), tANK6’s (green squares), and cANK6r’s (blue triangles) binding to the plasma proteins human serum albumin (HSA) and to *α*1 acid glycoprotein (AGP) was analyzed. The D-peptides were applied at 5*μ*M while HSA and AGP concentrations were adjusted as follows: HSA 7.4*μ*M to 420*μ*M, AGP 0.04*μ*M to 3*μ*M. The unbound amount of ANK6, tANK6, and cANK6r (in %) to HSA or AGP respectively was plotted against the D-peptides’ concentrations. Datasets were fitted by nonlinear regression to determine the K_D_.

Preclinical pharmacokinetic investigations were performed with ANK6, tANK6, and cANK6r *in vitro* and *in vivo*. First, affinity to the two most recurrent human plasma proteins, HSA and AGP, were determined in order to estimate the D-peptides’ PPB and the resulting fractions unbound (f_u_) in plasma after oral administration. Results revealed about 2000 times higher affinities of all three D-peptides to AGP (K_D_: ANK6 0.29*μ*M, tANK6 0.06*μ*M, cANK6r 0.28*μ*M) as compared to HSA (K_D_: ANK6 not analyzable, tANK6 137.7*μ*M, cANK6r 598.6*μ*M) (Fig. 4). The obtained affinities to HSA and AGP were used for prediction of the D-peptides’ overall f_u_ in plasma (1.43% ANK6, 0.29% tANK6, 1.36% cANK6r).

**Fig. 5.**
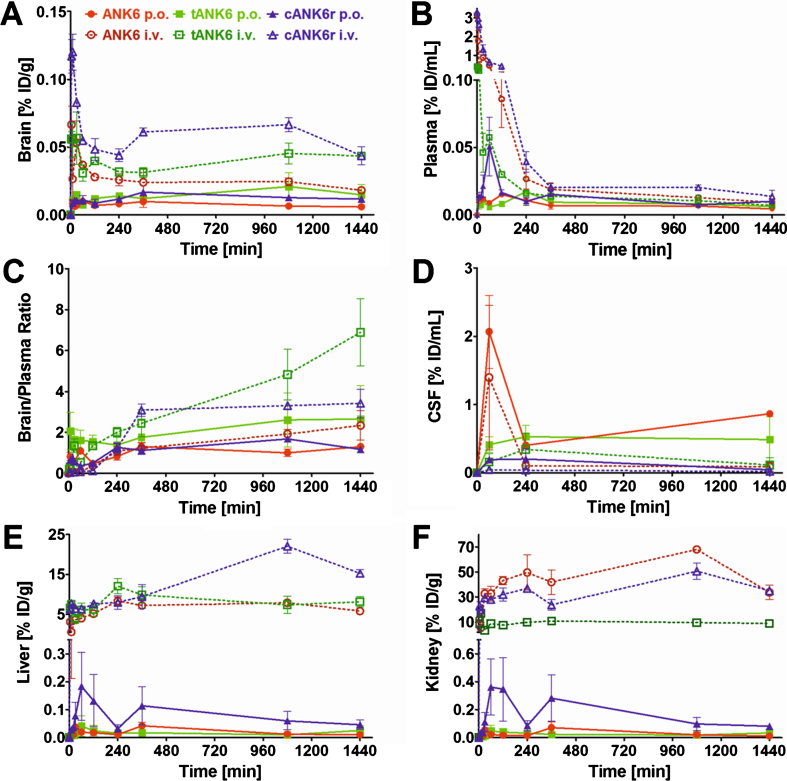
Pharmacokinetic concentration-time profiles of ANK6, tANK6, and cANK6r. Pharmacokinetic concentration-time profiles of ANK6 (red circles), tANK6 (green squares), and cANK6r (blue triangles) were investigated in brain (A), plasma (B), CSF (D), liver (E), and kidney (F) after i.v. (3.3 mg/kg, dotted lines) and p.o. (10 mg/kg ANK6 & tANK6, 15 mg/kg cANK6r, continuous lines) administration to wild type mice (3 mice/time point). The D-peptides were administered as a mixture of ^3^H-labelled and non-labelled peptide in 0.9% sodium chloride solution. The ^3^H-peptides’ concentrations (triplicate) in the respective organs, CSF, and plasma were measured with liquid scintillation counting. Total peptide concentrations were calculated as % of the injected dose per g or mL (% ID/g for brain, liver, kidney; % ID/mL for plasma, CSF) and plotted over time. The brain and plasma concentrations were set in relation to each other and plotted against the time as the brain/plasma ratio (C).

Since we have shown that ANK6, tANK6, and cANK6r inhibit A*β*_1 - 42_ aggregation, eliminate toxic A*β* oligomers, and lower A*β*_1 - 42_-induced cytotoxicity, we conducted pharmacokinetic studies of these peptides via two different administration routes, intravenous (i.v.) and oral (p.o.). Concentration-time profiles of brain, plasma, liver, and kidney are presented in Fig. 5A, B, E, and F while the condensed CSF concentration-time profiles for four time points are shown in Fig. 5D. The highest D-peptide concentrations relative to the injected dose per gram organ or milliliter plasma (% ID/g, % ID/mL) were detected in liver and kidney (organs of metabolization and elimination) followed by plasma (distribution), CSF, and the brain (site of action). As i.v. administration was followed by high initial plasma concentration peaks, the amount of D-peptides detected in liver and kidney from the first to the last measured time point (AUC [min*% ID/g]) was much higher as compared to amounts detected after oral administration. From 6 h until 24 h after administration, plasma levels were in the same range independent from the administration route (0.005 to 0.02 % ID/mL). Brain levels were substantially higher after i.v. administration as compared to oral administration of the respective D-peptides at all investigated time points. Thus, higher initial plasma levels led to higher brain levels, but interestingly also for the time points when plasma levels had already equalized (>6 h). An exception from these differences for the respective administration routes, i.e., i.v. levels > p.o. levels, was observed in CSF. Here, D-peptide levels were, contrary to expectations, higher after oral administration than after i.v. administration. Extraordinary high CSF levels were observed for ANK6 about 60 min after both types of administration. 4 h after administration, CSF levels of ANK6 were again in the same range as those of tANK6 and cANK6r. Of note, the brain/plasma ratios were about one or even higher already 4 h after administration (Fig. 5C). 24 h after administration, the ratios were between 1.17 (cANK6r, p.o.) and 6.89 (tANK6, i.v.).

The maximum concentrations relative to the injected dose (C_max_, % ID/mL) in plasma increased from tANK6 (i.v. 0.40, p.o. 0.02) over ANK6 (i.v. 3.17, p.o. 0.02) to cANK6r (i.v. 3.29, p.o. 0.05) while in the brain, the C_max_ value (% ID/g) was dependent on the administration route. After i.v. administration, C_max_ increased from tANK6 (0.06) over ANK6 (0.07) to cANK6r (0.12). After oral administration, C_max_ increased from ANK6 (0.01) to tANK6 (0.02) and cANK6r (0.02).

The pharmacokinetic parameters in plasma and brain are summarized in Table 3. In plasma, compound exposure over time relative to the dose (AUC_ %ID,0 - 1440_, min*% ID/mL) after i.v. administration was highest for the cyclic 13-mer cANK6r (181) followed by the linear 12-mer ANK6 (91.7) and finally by the linear 24-mer tANK6 (26.1). After oral administration, AUC_ %ID,0 - 1440_ was still highest for cANK6r (17.9) but tANK6 (12.9) and ANK6 (10.8) changed the order whereby differences between the three D-peptides’ AUC_ %ID,0 - 1440_ were smaller after oral administration. These findings were also reflected in the terminal half-lives (t_1/2_) which were determined to be longer after oral (22–31 h) than after i.v. (15–18 h) administration. Consistently, terminal clearance (CL, mL/(min*kg)) was lowest for cANK6r (i.v. 18.4), followed by ANK6 (i.v. 32.0), and tANK6 (i.v. 88.9). Oral bioavailability (F) was calculated to be 9.9% for cANK6r, 11.8% for ANK6, and 49.4% for tANK6. Interestingly, the mean residence time (MRT) had the same order within the three D-peptides (tANK6 > ANK6 > cANK6r) for both administration routes. Overall, the MRT values were higher after oral administration (9–11 h) than after i.v. administration (2–7 h).

**Table 3:** Pharmacokinetic parameters for ANK6, tANK6, and cANK6r in murine plasma and brain

PLASMA
**D**-peptide	**ANK6**	**tANK6**	**cANK6r**	**ANK6**	**tANK6**	**cANK6r**
**administration route**	**i.v.**	**i.v.**	**i.v.**	**p.o.**	**p.o.**	**p.o.**
**parameter**	**unit**
D	mg/kg	3.3	3.3	3.3	10	10	15
C_max_	% ID/mL	3.17	0.40	3.29	0.02	0.02	0.05
t_max_	min	0	0	0	120	240	60
AUC_ %ID,0 - 1440_	min*% ID/mL	91.7	26.1	181.0	10.8	12.9	17.9
MRT	h	3	7	2	10	11	9
*λ*_z_	1/min	0.00077	0.00063	0.00064	0.00054	0.00038	0.00039
t_1/2_	h	15	18	18	22	31	30
F (AUC_ %ID_)	%	na	na	na	11.8	49.4	9.9
CL	mL/(min*kg)	32.0	88.9	18.4	174.5	113.0	135.5
BRAIN
**D-peptide**	**ANK6**	**tANK6**	**cANK6r**	**ANK6**	**tANK6**	**cANK6r**
**administration route**	**i.v.**	**i.v.**	**i.v.**	**p.o.**	**p.o.**	**p.o.**
**parameter**	**unit**
C_max_	% ID/g	0.07	0.06	0.12	0.01	0.02	0.02
t_max_	min	5	30	5	60	1080	360
AUC_ %ID,0 - 1440_	min*% ID/g	35.8	56.6	85.6	11.0	22.5	19.1

In the brain, AUC_ %ID,0 - 1440_ (min*% ID/g) after i.v. administration for cANK6r (85.6) was higher than for tANK6 (56.6), and for ANK6 (35.8). In contrast to the plasma values after oral administration, tANK6 (22.5) showed the highest AUC_ %ID,0 - 1440_ followed by cANK6r (19.1) and ANK6 (11.0) in the brain. MRT in the brain was similar for all three D-peptides (11–14 h) independent from the respective administration route.

Calculated values of four commonly used BBB parameters, determined to allow for global comparison of any peptides’ efficiencies to cross the BBB, are listed in Table 4. The logBB value describes the blood-brain equilibrium distribution: negative logBB values result from lower AUCs in the brain than in plasma, whereas positive logBB values result from higher AUCs in the brain than in plasma. K_in_ describes the BBB permeability kinetics while V_i_ describes a peptides’ fictional (initial) distribution volume in the brain. The PS represents the uptake clearance from blood to brain. In this study, logBB values of ANK6 and cANK6r were below zero while tANK6’s logBB value was greater than zero. Regarding the graphically determined K_in_ and V_i_ values, ANK6 and cANK6r were in the same range while the values of tANK6 were increased by around factor 10. As PS was determined in consideration of a presumed CBF of 1.07 mL/(g*min) [32], values of all three D-peptides were the same as for K_in_.

**Table 4:** Blood-brain barrier values for ANK6, tANK6, and cANK6r after i.v. administration

Parameter	Unit	ANK6	tANK6	cANK6r
logBB	–	–0.401	0.335	–0.329
K_in_	mL/(g*min)	0.0003	0.0016	0.0003
V_in_	mL/g	0.0575	0.5833	0.0396
PS	mL/(g*min)	0.0003	0.0016	0.0003

## DISCUSSION

The approach of A*β* oligomer elimination by D-peptides clearly differs as compared to, e.g., antibodies directed against A*β*, or *β*- or *γ*-secretase inhibitors that already failed in several clinical trials. As opposed to passive immunization, the D-peptides’ therapeutic efficacy is independent from the individual immune system, and they are able to destroy already existing A*β* oligomers, a property which makes them superior to secretase inhibitors. The most obvious reason why numerous A*β* antibodies have failed in clinical trials is most likely that they had been either directed against A*β* fibrils or monomers, which are the wrong targets, or do bind to all forms of A*β* assemblies. In contrast, the results published so far for the A*β* oligomer targeting antibody Aducanumab are very promising and do underline the reasonability of the therapeutic approach of our A*β* oligomer eliminating D-peptides [33]. Consequently, the D-peptides could already show very promising results of therapeutic *in vivo* studies in various transgenic AD mouse models [10, 12, 15, 17, 19, 27, 34, 35].

In this study, we focused on optimizing ANK6 regarding its potency to eliminate toxic A*β* oligomers as well as its pharmacokinetic *in vivo* characteristics by designing two derivatives. To increase the D-peptide’s binding avidity to A*β*, so-called tandem peptides were developed and investigated before [19, 26]. They usually are head-to-tail juxtapositions of two 12-mer D-peptides resulting in a linear 24-residue D-peptide. Thus, tANK6 was included in this study to find out whether a tandem version indeed showed enhanced A*β*-targeting potency as compared to single ANK6. Another optimization approach, which aimed to increase blood-brain barrier permeation, revealed the 13-mer cANK6r. Previously, it had been shown that cyclic isoforms of several D3-derivatives reached remarkably higher brain levels after administration to wild type mice as compared to the corresponding linear peptides [25]. Consequently, we included cANK6r in this study to find out whether cyclization had an impact on ANK6’s *in vitro* potency and whether cyclization, here, also led to higher brain levels in comparison to the originally selected linear peptide although it contains six amino acid residue substitutions as compared to D3.

The comparison of *in vitro* properties of ANK6, tANK6, and cANK6r included different experimental approaches, namely A*β* aggregation, cell viability, and QIAD assays. Thereby we could validate our assumption that tANK6 has an enhanced potency as it most efficiently inhibited the A*β*_1 - 42_ fibril formation with a resulting IC_50_ of 2.1*μ*M as compared to ANK6 (3.6*μ*M), cANK6r (3.7*μ*M), and D3 (7.2*μ*M). Additionally, one could observe that only tANK6 and ANK6 did completely inhibit A*β*_1 - 42_ fibril formation by two-fold molar excess with regard to A*β* whereas cANK6r and D3 needed about eight-fold molar excess. Cell viability assays, which were conducted to investigate the D-peptides’ efficiencies to reduce A*β*-induced cytotoxicity in PC12 cells, showed a similar trend: tANK6 was most efficient with an IC_50_ value of 4.4*μ*M followed by ANK6 (11*μ*M) and cANK6r (14*μ*M). Although this experiment might suggest that supra-stoichiometric ratios of the D-peptides as compared to A*β* were required to demonstrate beneficial effects on A*β* aggregate cytotoxicity, one has to keep in mind that the D-peptides were added to the cell cultures together with preformed A*β* aggregates. Thus, the outcome of the experiment depends not only on the absolute concentrations and ratios of concentration, but also on incubation times. It was, however, not the scope of this more pharmacokinetic investigation to explore the kinetics of A*β* aggregate toxicity reduction in detail, but rather to compare efficacies among the D-peptides under one defined condition. PC12 cells were used for this experiment because they had originally been established by Greene and Tischler for “neurobiological and neurochemical studies” [36] and are nowadays a commonly used cell line in AD research [37, 38]. Additionally, the MTT test with PC12 cells belongs to our standard test battery for newly developed D-peptides to allow for comparison with data generated previously [10, 27]. The most meaningful *in vitro* assay QIAD awarded all three investigated D-peptides very promising oligomer eliminating characteristics as they drastically eliminated A*β* oligomers (>96% oligomer reduction in fractions 4–6). Previously published data for D3, generated in this test with exactly the same setting, show that D3 could, in the same molar ratio of A*β*:D-peptide as used in this study, reduce the A*β* oligomers by 51% [34]. Because we have developed the ANK compounds for their ability to stabilize A*β* monomers in an aggregation-incompetent conformation, one would expect to see an increase of the A*β* content in the monomer containing fractions (1 and 2). However, as already observed and described with D3 and its derivative D3D3, under artificially high *μ*M concentrations of A*β* and compound, the limited solubility of A*β* leads to high molecular weight co-precipitates composed of compound and A*β*. These co-precipitates do not have any property of A*β* oligomers and have previously been characterized and shown to be non-toxic, non-amyloidogenic, amorphous and ThT-negative [12]. In any case, all three compounds were able to eliminate the A*β* oligomers and converted them into non-toxic co-precipitates as was already shown previously for the lead compound D3 [12, 24] and another D3 derivative, RD2 [34].

In the following, PPB experiments were conducted to predict the D-peptides’ affinities to the most abundant human plasma proteins, HSA and AGP. If a drug strongly binds to plasma proteins, it is possible that the drug is in the organism but is not able to leave the circulation to the site of action. For AD drugs, this could mean that the drug circulates in plasma, but does not reach the brain, at least not to a very high proportion. Conversely, one can make use of PPB to a certain extent as there is always a dynamic equilibrium between drug bound and freely circulating in plasma (f_u_). Thus, plasma proteins can work as kind of drug releasing depots leading to consistent drug distribution in plasma over time. This can lower the risk of adverse side effects and at the same time lead to longer MRTs [39, 40]. Slow drug release from the plasma proteins might even also allow for once daily drug administration, which is followed by the best patients’ compliance [41].

ANK6, tANK6, as well as cANK6r bound to AGP (K_D_ between 0.06 and 0.29*μ*M) much stronger than to HSA (K_D_ of 138*μ*M and higher). This had been expected before as all three D-peptides have a positive net charge and AGP is known to more strongly bind positively charged molecules as compared to HSA, which is known to bind rather acidic or neutral molecules [40]. This had been observed previously for D3 in a similar manner. Jiang et al. determined a K_D_ of 1.8*μ*M for D3 to AGP while the K_D_ of D3 to AGP was above the detection limit of the used kit (>1.4 mM) [20]. The unbound fractions (f_u_) of the three D-peptides regarding PPB to HSA and AGP were determined to be in the same range (0.29–1.43%). Results again confirmed the assumption that tANK6 (f_u_ 0.29%) bound strongest to AGP because it consists of twice as many basic amino acid residues as compared to ANK6 and cANK6r.

Summarizing the *in vitro* studies, cANK6r’s slight inferiorities after the A*β* aggregation inhibition and cell viability tests, possibly caused by structural hindrances due to cyclization, could be balanced by cANK6r’s very beneficial QIAD outcome in eliminating A*β* oligomers while not strongly affecting A*β* monomer levels. Nevertheless, tANK6 showed very promising results in all conducted *in vitro* tests, especially supporting the hypothesis that our tandem peptides do possess enhanced A*β*-targeting efficacy *in vitro*. As compared to the lead structure D3, the ANK peptides investigated in this study do show the same or, especially in the most important *in vitro* QIAD assay, even more promising results in these A*β* interaction assays. This is why we consider these D-peptides, especially tANK6 and cANK6r, very promising A*β*-targeting D-peptides which are supposed to be therapeutically active even if they reach lower brain levels than D3. One example for the importance of high efficacy, as determined *in vitro*, is the tandem D-peptide D3D3, which has demonstrated already higher efficacy than D3 *in vivo*, despite lower brain penetrance [19].

After extensive *in vitro* investigation of the three D-peptides, they were pharmacokinetically investigated in further detail. For these studies, a mixture of ^3^H-labelled and non-labelled D-peptide was administered to C57Bl/6N wild type mice. On the one hand, we used C57Bl/6N mice as it is the gold standard to conduct pharmacokinetic experiments with new compounds in young, healthy wild type organisms, especially in rodents [42, 43]. On the other hand, we used exactly these mice at this age in order to allow for direct comparison to our results of D3 and other D3-derivatives that had been determined before using exactly the same experimental method [20, 26, 29]. As D-peptides had been proven to be extraordinary stable against proteolytic degradation and metabolites can therefore be neglected, the detected amount of ^3^H-labelled D-peptide in the murine samples (brain, plasma, liver, and kidney) was anticipated to correctly reflect the total D-peptide concentration. This has been described and evaluated several times before [20, 25, 26, 29]. As each D-peptide’s ^3^H-label was located in a leucine alkyl group (4,5-^3^H-D-Leu), the labels were also considered to be biologically stable [25]. Further underlining the D-peptides’ stabilities, Elfgen et al. confirmed this by incubation in several media simulating the oral administration route with HPLC analyses [22]. Thus, the amount of ^3^H-labelled D-peptide quantified in LSC measurements was used for back-calculation of the total D-peptide concentration in the respective samples and pharmacokinetic parameters were calculated subsequently. However, it cannot be ruled out completely that all potential minor modifications of the tritium-labelled compounds could be detected by the applied methods. Thus, we cannot be completely sure that radioactivity correlates absolutely 100% with unmodified compound. This certainly is a limitation of the study that needs to be taken into account and should be stated here clearly for the sake of scientific rigor.

Apparently, the pharmacokinetic profiles of liver and kidney revealed huge differences after i.v. and oral administration. The fact that i.v. administration was followed by far higher D-peptide levels in the organs responsible for metabolization and excretion could be explained by the initially higher plasma levels. After the rapid initial rise, liver and kidney levels remained quite constant throughout the observation time of 24 h. Thus, one could surmise that the D-peptides were not immediately excreted but that they accumulated in liver and kidney for some time. Although the administered dose was three times higher for oral than for i.v. administration, only relatively small amounts of the D-peptides were taken up via the gastrointestinal tract. Direct comparison to data of D3 (generated by Jiang et al. using exactly the same experimental setup before [20]), revealed that plasma levels of ANK6, tANK6, and cANK6r were considerably lower especially in plasma after oral administration. Regarding the brain, the AUCs of D3 after oral administration were 7.2 (tANK6), 7.7 (cANK6r), and 13.4 (ANK6) times higher as compared to the respective D-peptides. Thus, the notable differences in plasma and consequently also in brain levels between D3, and ANK6 and its derivatives most likely derived from the different amino acid residue compositions. This assumption is substantiated by the fact that brain and plasma levels of other D3-derivatives, composed of the same amino acid residues as D3, were rather in comparable ranges to those of D3 than to those of ANK6 and its derivatives [20, 25, 26]. These pharmacokinetic findings might also partially explain why the intraperitoneal treatment study (4 weeks) with ANK6, conducted by Klein et al., only showed “a non-significant tendency for improving memory performance of tg-APPSwDI mice” [23]. Despite the fact that ANK6 and its derivatives do reach lower brain levels as compared to their lead compound D3, we still consider tANK6 and cANK6r very promising drug candidates for future therapeutic studies because of their superior A*β* interaction *in vitro* efficiencies.

Probably, huge parts of orally administered ANK6, tANK6, and cANK6r in this pharmacokinetic study were immediately excreted. Interestingly, plasma levels after i.v. and oral administration were in the same range about 6 h after administration while brain levels of the i.v. administered D-peptides, especially those of cANK6r and tANK6, remained higher than those of the orally administered D-peptides. These findings suggested that the D-peptides’ outward transport across the BBB, back into plasma, was not as fast as plasma clearance. A reason for that might have been that the D-peptides bound to structures in the murine brain or that the outward transport was limited. Either way is beneficial for an AD drug candidate as the brain is supposed to be its site of action. In this context, the cyclic peptide (cANK6r) revealed higher brain levels than its linear equivalent (ANK6) or the tandem D-peptide (tANK6) after i.v. administration, again substantiating the previously set up hypothesis that cyclization results in more efficient BBB permeation [25]. Finally, regarding the C_max_ values in plasma as well as in brain for both administration routes, cANK6r always revealed the highest values suggesting and confirming high stability and enhanced abilities to cross membranes.

Regarding pharmacokinetic parameters in plasma disclosed a bigger difference within the AUC_ %ID,0 - 1440_ values after i.v. than after oral administration, most likely due to different uptake and accumulation characteristics in liver and kidney. These characteristics had more impact after i.v. administration, as plasma levels were initially higher. After oral administration, when most of the administered D-peptides had already been eliminated due to the first-pass effect, the impact of liver and kidney over time was smaller. The unexpected result that tANK6 revealed the highest oral bioavailability might be relativized by consideration that tANK6 had a relatively low plasma AUC_ %ID,0 - 1440_ after i.v. administration in contrast to the two other peptides. Thus, tANK6’s oral bioavailability was higher than those of ANK6 and cANK6r despite the fact that their plasma AUC_ %ID,0 - 1440_ after oral administration were in a similar range. Conversely, cANK6r’s unexpected low bioavailability was explained by the same approach. Regarding t_1/2_, a therapeutic regimen with once daily dosing would be applicable as the values varied between 15 and 31 h. During a therapeutic study, the steady state levels would be attained after 3 to 6.5 days as a general rule states that the steady state is reached after five t_1/2_ periods [44]. As t_1/2_ for ANK6 was determined to be shorter than for tANK6 and cANK6r, the two derivatives could be awarded being more favorable regarding t_1/2_. Comparison of i.v. and oral administration generally pointed out longer t_1/2_ after oral administration. These findings were in accordance with the general opinion that long-term AD-treatment in elderly people would be done best orally, not least because of the small impact on the patients’ daily living.

To allow for comparability to other peptides listed in “Brainpeps: the blood-brain barrier peptide database”, four characterizing BBB values were determined (Table 4) [31]. tANK6’s logBB value (positive sign) reflected that the peptide’s drug exposure over time was greater in the brain than in plasma whereas for ANK6 and cANK6r (negative signs) it was the other way around. Thus, tANK6r seemed to have entered the brain from plasma most efficiently. However, one may not neglect that logBB values depend on binding to plasma and brain tissue as well as on active transport. K_in_ and V_i_ had been graphically determined with regard to investigate the velocity of the three D-peptides’ BBB passage. Remarkably, both values were tenfold greater for tANK6 than for ANK6, and cANK6r underlining that tANK6 crossed the BBB fastest. Uptake clearance from blood to brain was calculated based on K_in_ so that tANK6 here also revealed the highest PS value. To classify ANK6 and its two derivatives, their BBB parameters were compared to those of Dermorphin, a potent natural opioid consisting of seven amino acid residues including one D-enantiomeric amino acid residue. Dermorphin had been suggested as positive control [31]. Dermorphin’s K_in_ values were determined to be between 0.0002 and 0.0022 mL/(g*min) while its V_i_ values were determined to be between 0.0162 and 0.0215 mL/g [45, 46]. By all means, especially tANK6’s, but also ANK6’s and cANK6r’s, BBB parameters were in the same scale as Dermorphin’s awarding the three D-peptides a very efficient BBB permeability.

Summarized, ANK6 and its two derivatives, tANK6 and cANK6r, showed very beneficial A*β*-targeting *in vitro* efficacies. Analyzing the results, it became obvious that both newly developed ANK6-derivatives, tANK6 and cANK6r, had superior A*β* interacting properties as compared to ANK6. As shown already for ANK6’s predecessor peptide D3, i.v. administration led to accumulation in liver and kidney, whereas p.o. administration did not [20]. Oral bioavailabilities were with about 10% for ANK6 and cANK6r and 50% for tANK6 very high when compared with typical oral bioavailabilities of I-enantiomeric peptides. cANK6r showed the highest drug exposure over time in the brain. All three investigated compounds revealed very high BBB penetration as indicated by typically determined BBB values (Table 4).
